# Conspiracy thinking, belief in false information about COVID-19 and the severity of anxiety and depression symptoms in Polish and Spanish respondents in the final phase of the COVID-19 pandemic - preliminary report from a comparative study

**DOI:** 10.1192/j.eurpsy.2025.462

**Published:** 2025-08-26

**Authors:** P. Dębski, A. Cetnarowska, W. Madejczyk, E. M. Benito, M. Piegza, J. Smolarczyk, J. Blanch

**Affiliations:** 1 Department of Psychiatry, Medical University of Silesia, Tarnowskie Góry; 2 Institute of Psychology, Humanitas University, Sosnowiec, Poland; 3 Department of Psychiatry, Hospital Universitari de Santa Maria, Lleida, Spain; 4 Student Scientific Association, Medical University of Silesia, Tarnowskie Góry, Poland; 5 Faculty of Education, Psychology and Social Work, University of Lleida, Lleida, Spain; 6 Department of Psychoprophylaxis, Medical University of Silesia, Tarnowskie Góry, Poland; 7 Director of Mental Health and Addiction Services, Hospital Universitari de Santa Maria de Lleida, Lleida; 8 CIBERSAM, Madrid, Spain

## Abstract

**Introduction:**

Conspiracy thinking refers to the tendency to accept explanations about reality that are different from those that are most rational or best proven. This trend became a significant threat during the COVID-19 pandemic, as belief in false information about COVID-19 was an important factor inhibiting preventive actions. The tendency to think conspiratorially has also been associated with the severity of some psychopathological symptoms, such as anxiety and depressive symptoms.

**Objectives:**

The aim of the study was to assess the differences in general and COVID-19 conspiracy thinking between Polish and Spanish respondents, as well as the relationship between conspiracy thinking and the severity of anxiety and depression symptoms.

**Methods:**

192 Polish citizens (average age of 39.7 ± 8.61 years; women: 151, men: 41) and 190 Spanish citizens (average age of 48.3 ± 10.03 years; women: 139, men: 51) were recruted. The GCBS (Brotheron, et al.), the COVID-19 CBS (Dębski, et al.) and the HADS (Zigmond & Snaith) tools were used. The Shapiro-Wilk test, the Mann-Whitney U test and Spearman’s correlation coefficient were implemented.

**Results:**

In Spain, the highest average score in terms of conspiracy thinking about COVID-19 was obtained by the statement: “Governments deliberately spread false information about COVID-19 in order to conceal the actual state of the pandemic” (mean = 2.784), while in Poland this statement was: “SARS-CoV-2 tests are unreliable, they may be positive in the case of infection with another virus” (mean = 2.912). Spanish citizens obtained significantly higher scores than Poles on the Malevolent global conspiracies scale (median 9 vs 8, p=0.024), as well as in two false beliefs about COVID-19: “Wearing face masks causes oxygen deficiency…” (median 3 vs 2, p=0.000) and “There is a drug that can effectively cure COVID-19 patients, but information about it is confidential…” (median 3 vs 1.5, p =0.026). Among Polish citizens, the belief: “Health workers receive financial benefits for diagnosing COVID-19…” was higher (median 2 vs. 1, p=0.000). In the Spanish group, most of the GCBS subscales correlated positively with the anxiety and depression symptoms. COVID-19 CBS also correlated with anxiety. In the Polish group positive correlations were noted between two GCBS scales and the severity of depression symptoms. There were no significant associations between GCBS and COVID-19 CBS with the anxiety symptoms in that group.

**Conclusions:**

The group studied in Spain showed a significantly higher intensity of conspiracy beliefs in more issues than the group studied in Poland. At the same time, conspiracy thinking in the Spanish group showed more pronounced relationships with the severity of anxiety and depression symptoms than in the Polish group.

**Disclosure of Interest:**

None Declared

